# Deep Learning‐Based Multimodal Fusion of Whole‐Slide Images and RNA Sequencing Identifies Survival‐Relevant Glioblastoma Clusters

**DOI:** 10.1002/cam4.72182

**Published:** 2026-08-10

**Authors:** Amin Zadeh Shirazi, Guillermo A. Gomez

**Affiliations:** ^1^ Centre for Cancer Biology Adelaide University Adelaide South Australia Australia; ^2^ Department of Computer Engineering, Faculty of Engineering Khayyam University Mashhad Iran

**Keywords:** AI, deep learning‐based multimodal fusion, glioblastoma, survival analysis

## Abstract

Glioblastoma is profoundly heterogeneous, and single‐modality analyses often miss prognostically relevant structure. We introduce a transparent, end‐to‐end workflow that fuses available whole‐slide histology and RNA‐seq to discover clinically meaningful glioblastoma subgroups using an unsupervised learning model after feature extraction. Haematoxylin–eosin slides are tiled, tissue‐screened and stain‐normalised; tiles are embedded with a pretrained ResNet‐50 to yield 2048‐dimensional features, averaged per patient and compressed to 30‐D by an autoencoder. In parallel, RNA‐seq (~48 k genes) undergoes low‐variance filtering and normalisation, then a second autoencoder produces a 30‐D transcriptomic embedding. The two 30‐D representations are concatenated into a 60‐D fused vector, robustly scaled and refined with PCA (≈98% variance retained). Across *K*‐means, Gaussian mixture models and Agglomerative clustering (*k* = 2–20), Agglomerative *k* = 2 was decisively best (mean silhouette ≈0.53), yielding clusters of 150 and 8 patients (survival subset 147 and 8). Survival separation was substantial (median 454 vs. 138 days; log‐rank *p* = 0.0096). In Cox models, the poorer‐prognosis cluster showed increased risk (HR ≈ 2.70), which remained significant after age adjustment (HR = 2.15, 95% CI 1.04–4.46; age per year HR = 1.02, 95% CI 1.01–1.04). Attribution and consensus analyses yielded compact, interpretable gene sets (22 shared; 8 per cluster), including markers associated with NOTCH/γ‐secretase and oxidative phosphorylation. These findings nominate biologically plausible hypotheses for future validation rather than immediate treatment‐selection rules. Overall, this study demonstrates that auditable late fusion of histology and transcriptomics, built from routine data, can identify survival‐associated glioblastoma subgroups and provides a hypothesis‐generating framework for prospective, harmonised, multi‐centre validation.

## 1 Introduction

Glioblastoma is the most aggressive primary malignant brain tumour in adults, with standard chemoradiotherapy yielding a median overall survival of roughly 15 months, highlighting the need for more precise, biology‐grounded patient stratification [1]. Large consortia have established that glioblastoma is profoundly heterogeneous at the genomic and transcriptomic levels and can be partitioned into clinically relevant expression subtypes, each enriched for distinct driver alterations [2, 3] This molecular diversity is mirrored by variability in routine haematoxylin–eosin (H&E) histomorphology, motivating joint analysis of tissue architecture and gene expression. Digital pathology enables population‐scale interrogation of whole‐slide images (WSIs). Weakly supervised deep learning has delivered clinical‐grade diagnostic performance on WSIs without pixel‐level annotations, demonstrating that slides encode rich prognostic signals [4]. Moreover, convolutional neural networks can infer tumour genotype directly from H&E, indicating that morphology recapitulates molecular programmes [5]. Robust pipelines must also address inter‐laboratory stain variation; structure‐preserving normalisation via sparse colour deconvolution is widely adopted in computational pathology [6]. On the transcriptomic side, RNA‐seq provides tens of thousands of features per patient; non‐linear representation learning is therefore essential. Denoising autoencoders construct compact embeddings that retain clinically meaningful signal from high‐dimensional expression data [7], while deep count autoencoders explicitly model over‐dispersion in RNA‐seq to produce stable latent spaces for downstream analysis [8].

Integrating modalities typically improves stratification over unimodal baselines. Unsupervised factor models such as MOFA reveal shared and modality‐specific axes of variation that are not apparent within single data streams [9]. In glioblastoma and other brain tumours, multimodal intelligence has already shown clinical promise: survival CNNs that combine histology and genomics outperform pathology‐only or genomics‐only models for overall‐survival prediction [10]. Attention‐based fusion of histology with omics has improved prognosis modelling in TCGA cohorts [11]. Recent multi‐cohort studies in brain cancers report that models combining histopathology with molecular profiles generalise better than unimodal approaches [12, 13]. Building on our own contributions, we have previously shown that deep learning on WSIs and transcriptomics yields clinically relevant signal: DeepSurvNet classified brain‐cancer survival from H&E WSIs, demonstrating that morphology encodes prognostic information [14]; GLIOBLASTOMA_WSSM, a dedicated CNN for whole‐slide segmentation, quantified tumour–perivascular niche interactions linked to poor outcome, establishing that spatial context in WSIs is prognostically informative [15]; DeeP4med learned robust transcriptomic representations to predict normal versus cancer expression profiles across multiple tissues, evidencing scalable feature learning from high‐dimensional RNA‐seq [16]. These studies validate our pipelines for (i) slide curation and feature extraction, (ii) survival‐relevant morphology modelling and (iii) transcriptomic representation learning; the present work unifies these strengths in a transparent late‐fusion framework that discovers biologically coherent, prognostically distinct glioblastoma subgroups.

**FIGURE 1:**
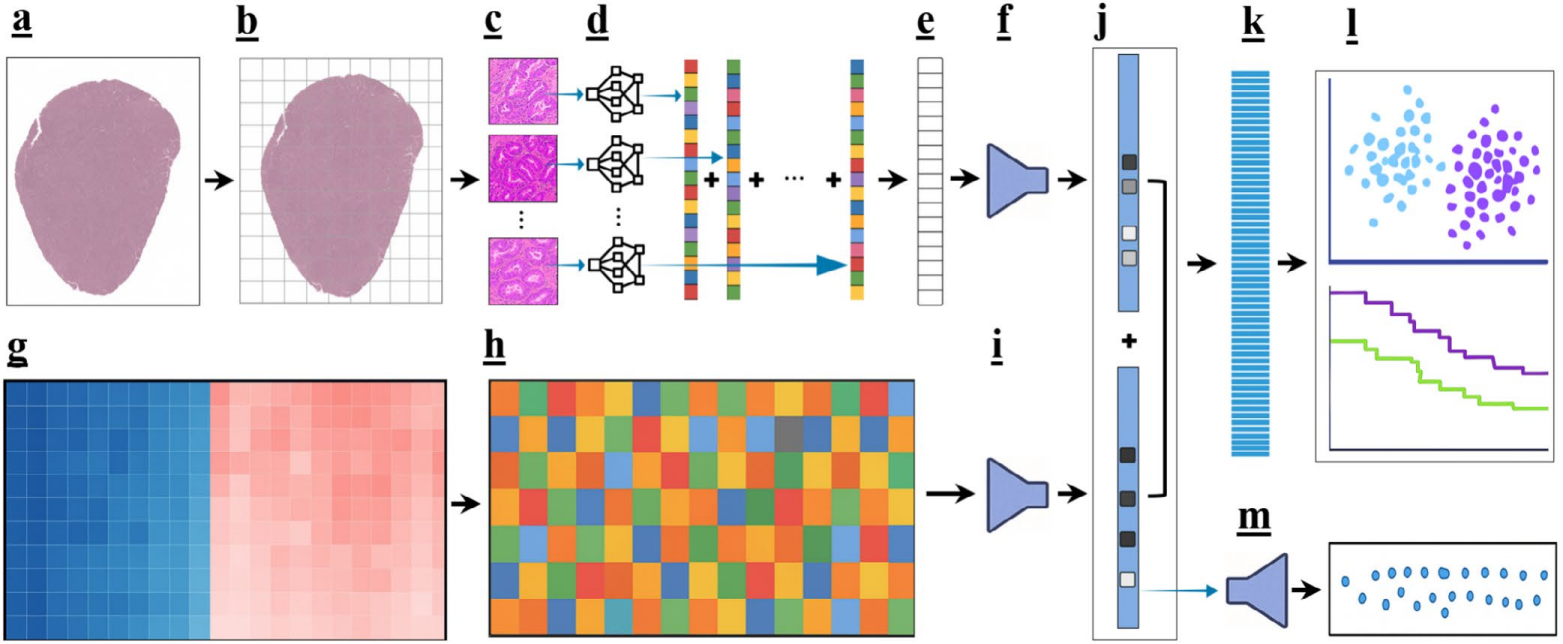
Multimodal workflow integrating histology and RNA‐seq for patient stratification. (a) Whole‐slide image (WSI) acquisition. (b) WSI tiling into non‐overlapping patches (20×). (c) Tissue‐containing tiles retained after quality control. (d) Patch‐level CNN feature extraction with a pretrained ResNet‐50 (2048‐D) and per‐patient aggregation. (e) Patient‐level histology feature vector (2048‐D). (f) Autoencoder (AE) compression of histology features to a 30‐D latent embedding. (g) RNA‐seq preprocessing with low‐variance gene cut‐off and normalisation. (h) Normalised gene‐expression matrix across patients. (i) AE compression of RNA‐seq (~48 k genes) to a 30‐D latent embedding. (j) Late fusion of the two feature representations obtained from histology (30‐D) and RNA (30‐D). (k) Final 60‐D multimodal feature vector per patient. (l) Clustering followed by survival analyses (Kaplan Meier and Cox) identify prognostic subgroups. (m) Discovery of high‐importance genes from RNA representations (30‐D) contributing to cluster separation and survival differences.

In this paper, we address this gap by providing a streamlined, fully reproducible workflow that fuses WSIs with RNA‐seq to discover multimodal GLIOBLASTOMA subgroups, using only unsupervised learning after feature extraction (Figure 1). Whole‐slide images are tiled (patches), tissue‐screened and stain‐normalised; tile features are extracted with a pretrained ResNet‐50 (2048‐D), averaged per patient and compressed to 30‐D via an autoencoder. In parallel, RNA‐seq (~48 k genes) is filtered for low variance, normalised and embedded to 30‐D with a second autoencoder. The two 30‐D representations are concatenated (late fusion) into a 60‐D patient vector, robustly scaled and refined by PCA, then clustered without supervision. This minimalist design (no task‐specific supervision or complex attention blocks) increases transparency and portability across centres. By mapping patients to multimodal subtypes together with compact, interpretable gene sets, the workflow offers a research‐grade route for generating hypotheses about risk stratification, pathway enrichment and future biomarker‐guided studies. Because all steps are codified and data‐agnostic, the approach can be extended to prospective and harmonised multi‐centre cohorts, where its clinical validity, robustness to technical heterogeneity and treatment‐response relevance can be formally tested.

## 2 Methods

### 2.1 Patient Cohort, Preprocessing and Construction of Patient‐Level Embeddings

We analysed 155 TCGA [17] glioblastoma cases with both whole‐slide images (WSIs) and bulk RNA‐seq available after quality control (QC); survival analyses in later sections are performed on the 147 versus 8 patients retained in each cluster after clinical curation (Figure 1 gives the overview).

### 2.2 WSI Preprocessing and Tile (Patch) Selection

Each WSI was processed at 20× magnification and divided into fixed‐size patches (tiles; 224 × 224 px). Tissue regions were identified using a hybrid thresholding mask, Otsu's method applied to RGB channels and to HSV saturation, combined with a minimum‐intensity guard and followed by binary closing (three dilations then three erosions) to remove small holes and speckles. This classical pipeline is robust for histology foreground detection and has long been used in digital pathology [18]. Tiles failing background (> 80% near‐white), low‐contrast or off‐tissue checks were excluded by QC logic. To reduce inter‐laboratory colour variability, all accepted tiles were stain‐normalised with the structure‐preserving method of Vahadane et al., which decomposes optical density into stain basis and concentrations and re‐projects concentrations onto a reference basis [6].

### 2.3 Tile‐Level Feature Extraction and Patient‐Level Histology Embedding

Each normalised tile was fed to a pretrained ResNet‐50 with the classification head removed to obtain a 2048‐dimensional feature vector per tile. Contemporary pathology foundation models trained at scale confirm that pretrained CNN/transformer features transfer strongly to histology, supporting our use of ImageNet‐initialised encoders for robust slide representations [19, 20]. Channel‐wise ImageNet normalisation (mean/SD) was applied for domain‐consistent inference. For every patient, tile features were averaged to produce a single 2048‐D histology vector—a simple aggregation that stabilises patient‐level estimates when tile counts vary.

### 2.4 RNA‐Seq Processing and Modality‐Specific Autoencoders

**FIGURE 2:**
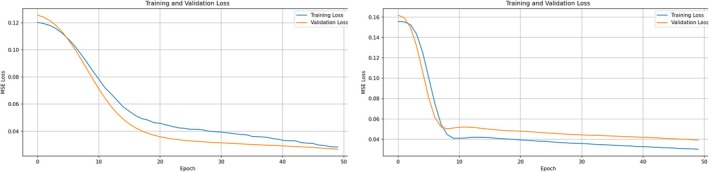
Autoencoder training phase. (Left) Histology AE training/validation loss across epochs; (right) RNA‐seq AE training/validation loss across epochs.

Bulk RNA‐seq (~48,000 genes per sample) was low‐variance filtered and normalised (Section 2), then passed to a denoising autoencoder that produced a 30‐dimensional transcriptomic embedding. In parallel, a second autoencoder compressed the 2048‐D histology vectors to 30‐D. Recent deep‐learning work demonstrates that autoencoder‐based latent spaces capture biologically meaningful signal from gene‐expression data and related omics, enabling compact, downstream‐ready embeddings [21, 22]. Training curves (Figure 2) showed smooth, monotonic decrease with minimal divergence between training and validation, indicating stable optimisation without overfitting. The two 30‐D embeddings were then concatenated (late fusion) into a 60‐D patient vector and robustly scaled. Principal component analysis (PCA) on the fused features retained ≈98% of the variance, yielding a low‐dimensional but information‐preserving space for unsupervised discovery.

## 3 Results

### 3.1 Multimodal Clustering and Model Selection

We benchmarked unsupervised clustering on four representations: (a) Histology‐only (30‐D AE embedding of WSI features), (b) RNA‐only (30‐D AE embedding), (c) fused RNA + Histology (60‐D; 30‐D per modality concatenated) and (d) fused RNA + Histology plus clinical covariates (age, sex; appended after robust scaling). For each representation, we compared Agglomerative (Ward linkage, Euclidean distance), *K*‐means and Gaussian Mixture Models (GMM) over *k* = 2–20, using the silhouette coefficient as the internal validity metric and Ward's minimum‐variance criterion for hierarchical clustering [23].

### 3.2 Histology‐Only (30‐D)

**FIGURE 3:**
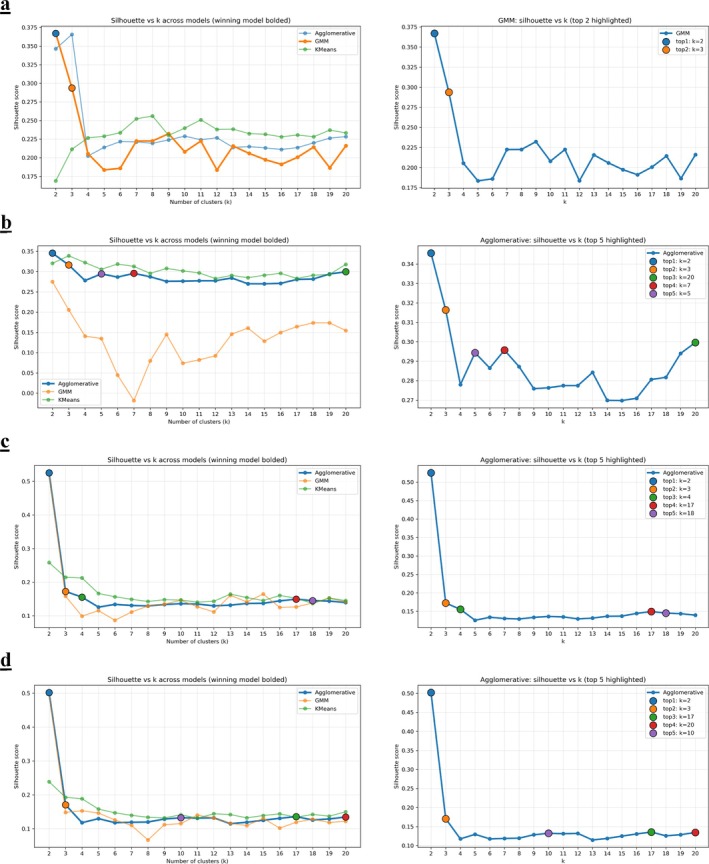
Silhouette‐based model selection across four representations and cluster numbers (*k* = 2–20). (a) (Histology‐only, 30‐D): (left) Silhouette versus *k* for Agglomerative (Ward–Euclidean), GMM and *K*‐means; (right) GMM focus with the top two *k* values marked. (b) (RNA‐only, 30‐D): (left) Model comparison as above; (right) Agglomerative focus with the top five *k* values highlighted (maximum at *k* = 2; silhouette ~0.30–0.35). (c) (RNA + Histology, 60‐D): (left) Model comparison showing clear dominance of Agglomerative; (right) Agglomerative focus with the top five *k* values highlighted (peak at *k* = 2, mean silhouette ≈ 0.53). (d) (RNA + Histology + Clinical): (left) Model comparison with age and sex appended; ranking unchanged relative to fused‐only; (right) Agglomerative focus with the top five *k* values highlighted (peak at *k* = 2, silhouette ≈ 0.50). Summary: The fused RNA + Histology embedding achieves the highest separation; adding age and sex does not materially change the optimal model or score.

Histology embeddings yielded moderate separation with the best scores at *k* = 2 (Agglomerative peak ≈ 0.34–0.36). *K*‐means performed similarly at small *k*, while GMM showed lower and less stable scores across *k* (Figure 3a). These results indicate that morphology alone contains prognostic structure, but not at the level achieved by multimodal fusion.

### 3.3 RNA‐Only (30‐D)

RNA embeddings alone produced modest separation (~0.30–0.35 at *k* = 2), with small differences between Agglomerative and *K*‐means and variable GMM performance (Figure 3b). This again suggests that either single modality is insufficient to fully resolve patient subgroups.

### 3.4 Fused RNA + Histology (60‐D)

Late fusion substantially improved cluster separability. Across models, Agglomerative delivered the highest separation at *k* = 2, with a mean silhouette ≈ 0.53, clearly outperforming *K*‐means and GMM at all *k* (Figure 3c). The elbow‐like drop beyond *k* = 2 indicates a parsimonious two‐group structure. We therefore select Agglomerative (Ward), *k* = 2 for downstream analyses; Ward's criterion is well‐suited to Euclidean, PCA‐refined embeddings and tends to form compact clusters [24]. Alternative cluster numbers, including *k* = 3, were evaluated within the same *k* = 2–20 model‐selection sweep. No higher‐*k* solution improved the internal validity relative to the Agglomerative *k* = 2 model, and the sharp decrease in silhouette beyond *k* = 2 supported retention of the simpler two‐cluster solution. We therefore interpret the *k* = 2 model as the most parsimonious representation of the fused RNA + histology embedding rather than as a definitive clinical subtype taxonomy.

### 3.5 Fused RNA + Histology + Clinical (Age, Sex)

To test whether simple clinical factors materially alter the latent structure, we concatenated age (*z*‐scored) and sex (binary) to the 60‐D fused vector. Model ranking and optimal *k* did not change: Agglomerative with *k* = 2 remained best with a silhouette ≈ 0.50, that is, within ~0.02 of the fused‐only setting (Figure 3d). Thus, the multimodal embedding already captures most separability; adding age and sex provides negligible incremental gain and does not perturb cluster selection.

In conclusion, across all four representations, the RNA + Histology fusion outperforms histology‐only and RNA‐only embeddings, and Agglomerative (Ward), *k* = 2 is the most parsimonious, best‐separated solution.

### 3.6 Two Candidate Subgroups in the Fused RNA–Histology Space

**FIGURE 4:**
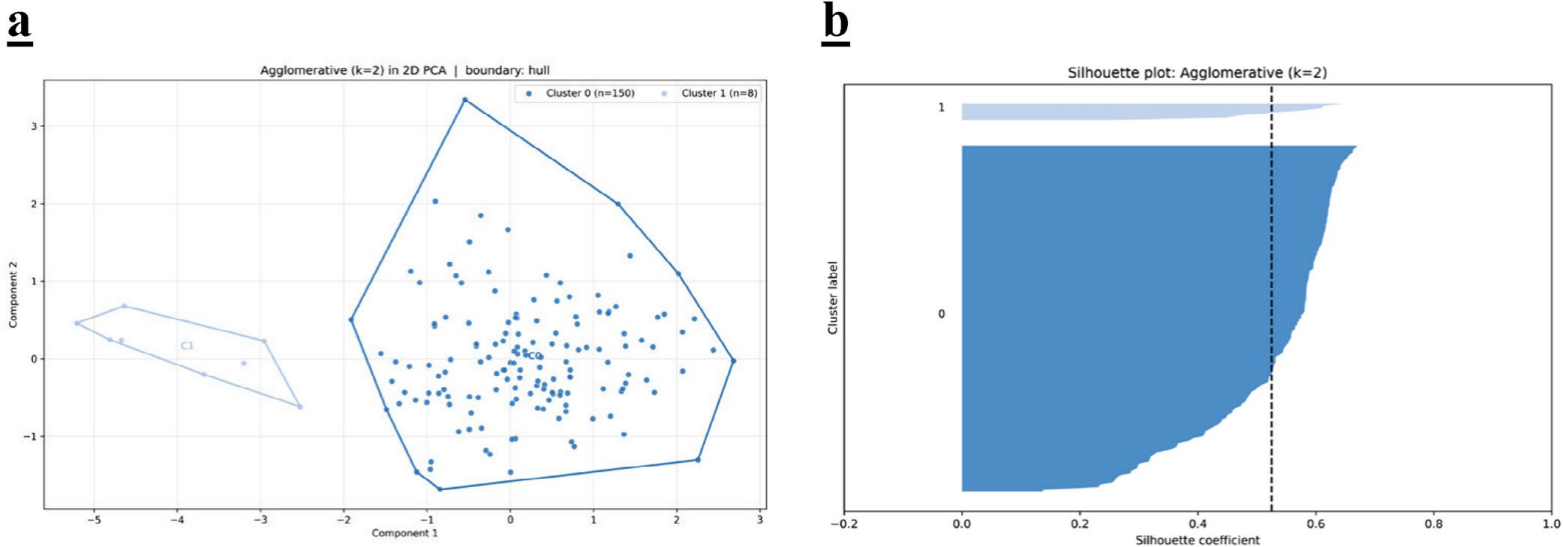
Fused RNA + histology clustering (Agglomerative, *k* = 2). (a) 2‐D PCA projection of the 60‐D fused embedding coloured by cluster; convex‐hull boundaries emphasise separation. Cluster sizes: C0 = 150, C1 = 8. (b) Silhouette plot for Agglomerative (*k* = 2); the dashed line marks the mean silhouette ≈0.53. Most samples have positive silhouettes and the minority cluster shows uniformly high values, supporting the selected two‐cluster solution for downstream survival analyses.

As shown in Figure 4, using the 60‐D fused embedding (30‐D histology + 30‐D RNA), Agglomerative clustering (Ward, Euclidean) with *k* = 2 produced the best separation among all models (mean silhouette ≈ 0.53). The solution yields a major cluster (*n* = 150) and a minor cluster (*n* = 8). A 2‐D PCA projection shows the minor cluster forming a compact group with a clear gap from the major cluster, whose boundary remains well confined, consistent with Ward's minimum‐variance criterion. The silhouette distribution indicates that the vast majority of samples have positive coefficients, with the minority cluster exhibiting the highest individual silhouettes, corroborating strong inter‐cluster separation and intra‐cluster cohesion. This two‐group solution is therefore carried forward to survival and Cox analyses. Given the small size of the minority cluster, this subgroup is interpreted as a candidate high‐risk phenotype requiring validation rather than as a clinically established glioblastoma subtype.

### 3.7 Survival Separation of the Fused RNA + Histology Subtypes

**FIGURE 5:**
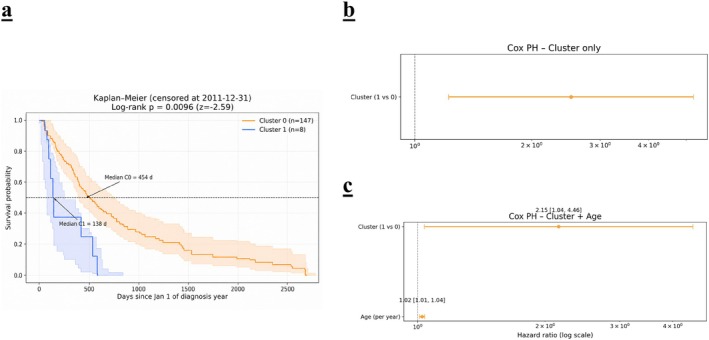
Survival impact of fused RNA + histology subtypes. (a) Kaplan–Meier survival curves comparing Cluster 0 (*n* = 147) and Cluster 1 (*n* = 8), derived from the 60‐D fused RNA + histology embedding. Survival time is shown as days since January 1 of the diagnosis year, with follow‐up censored at 2011‐12‐31. Solid step curves represent the estimated survival functions for each cluster, and shaded bands indicate pointwise 95% confidence intervals for the Kaplan–Meier survival estimates. Median survival times are indicated by the dashed horizontal reference line and arrow annotations: 454 days for Cluster 0 and 138 days for Cluster 1. The log‐rank test demonstrates significant survival separation between the two clusters (*p* = 0.0096, *z* = −2.59). (b) Cox proportional hazards model using cluster assignment only, showing increased hazard for Cluster 1 relative to Cluster 0: HR = 2.53, 95% CI 1.22–5.21. (c) Cox proportional hazards model adjusted for age, showing that Cluster 1 remains associated with increased hazard relative to Cluster 0: HR = 2.15, 95% CI 1.04–4.46; age is also independently associated with increased hazard per year: HR = 1.025, 95% CI 1.011–1.039. In panels b and c, the vertical dashed line indicates HR = 1, the *x*‐axis is log‐scaled and horizontal error bars denote 95% confidence intervals for Cox hazard‐ratio estimates.

The two clusters discovered in the 60‐D fused space show a marked difference in overall survival (Figure 5). Kaplan–Meier analysis (censoring at 2011‐12‐31) yields median survival 454 days for Cluster 0 versus 138 days for Cluster 1 (log‐rank *p* = 0.0096, *z* = −2.59). Thus, patients in the poorer‐prognosis cluster experience a reduction of ~316 days in median survival. Cox proportional hazards models (PHReg, Efron ties) confirm the effect size. In a cluster‐only model, Cluster 1 versus 0 is associated with a 2.53‐fold increase in hazard (HR = 2.53, 95% CI 1.22–5.21, *p* = 0.012). After adjusting for age, the cluster effect remains significant (HR 2.15, 95% CI 1.04–4.46, *p* = 0.039), and age adds independent risk (HR per year = 1.025, 95% CI 1.011–1.039, *p* = 4.98 × 10^−4^). These findings suggest that the multimodal clustering captures prognostic signal beyond age alone and identifies a small candidate high‐risk subgroup with substantially worse outcomes. However, because the poorer‐prognosis cluster contains only eight patients, the survival effect should be interpreted as hypothesis‐generating and requiring independent validation rather than as a clinically deployable subtype definition.

### 3.8 Gene Attribution From the RNA‐Seq Autoencoder Latent Space and Selection of the Top‐30 Markers

Rationale. To link the RNA autoencoder (AE) representation directly to individual genes, we quantified how much each input gene contributes to the 30‐dimensional latent vector. We used the Gradient × Input (Grad × Input) attribution used in our code: it is data‐aware (depends on the actual cohort values), architecture‐agnostic and provides a signed, unit‐consistent measure of input influence on each latent coordinate.

#### 3.8.1 Set‐Up

Let $x\in {\mathbb{R}}^{G}$ be the min–max scaled gene‐expression vector for a patient (genes as features, G≈48,000) and let the encoder $f:{\mathbb{R}}^{G}\to {\mathbb{R}}^{K}$ map x to the latent vector $z=f\left(x\right)$ with K=30.

#### 3.8.2 Per‐Gene, Per‐Latent Attribution (Grad × Input)

For each latent unit $k\in \left\{1,\ldots ,K\right\}$ and gene $g\in \left\{1,\ldots ,G\right\}$, the code computes

$$a_{\mathrm{gk}}\left(x\right)=x_{g}\cdot \frac{\partial z_{k}\left(x\right)}{\partial x_{g}},$$

Absolute attributions are then averaged over the cohort D(processed in batches) to obtain a stable, data‐level importance for the gene g on latent k:

$$A_{\mathrm{gk}}=\frac{1}{|D|}\sum_{x\in D}.|a_{\mathrm{gk}}\left(x\right)|.$$

#### 3.8.3 Global Gene Importance

To summarise across all latents, the code aggregates by an $L_{1}$–type sum:

$$\mathrm{GI}\left(g\right)=\sum_{k=1}^{K}.A_{\mathrm{gk}}.$$

This global importance score ranks genes by how strongly, on average, they influence the learned latent space.

#### 3.8.4 Selecting the ‘Top 30’ Markers for Visualisation

**FIGURE 6:**
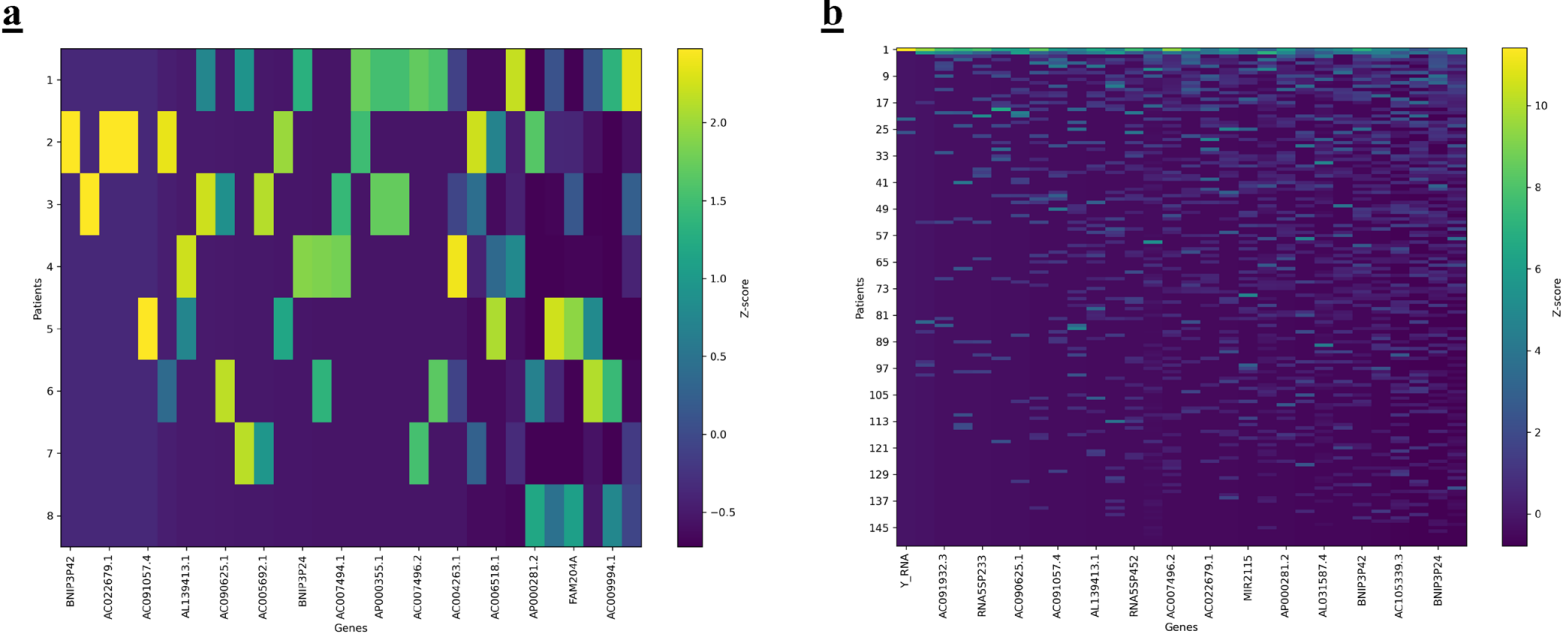
Cluster‐specific genes derived from the RNA autoencoder latent space. (a) (Cluster 1, n=8) shows the *z*‐scored expression of the top 30 Grad × Input genes, many of which display consistently elevated values across patients, indicating a compact expression programme aligned with the latent space that defines this poor‐prognosis subgroup. (b) (Cluster 0, n=150) presents the same 30 genes for the larger subgroup, with a more heterogeneous but still structured signal consistent with its broader dispersion in the fused embedding.

From Overall GradxInput, we retained the 30 highest‐ranked genes (largest $\mathrm{GI}\left(g\right)$). For clarity in the cluster heatmaps, expression values of these 30 genes were *z*‐scored per gene within the patient subset displayed and patients were ordered by their mean *z*‐score so that coherent patterns are visually apparent (Figure 6).

This procedure directly and quantitatively links the gene lists to the AE latent representation that underpins our clustering, ensuring that the markers highlighted in Figure 6a,b are those most responsible for shaping the transcriptomic latent space used in the multimodal discovery.

### 3.9 Consensus, Cluster‐Stable Gene Markers From RNA‐Seq

To derive cluster‐stable markers from the RNA modality, we applied a consensus filter directly to the expression matrix after clustering. For each cluster c with patient set $S_{c}$ and each gene g, expression values were *z*‐scored within the cluster,

$$z_{\mathrm{ig}}^{\left(c\right)}=\frac{x_{\mathrm{ig}}-\mu_{g}^{\left(c\right)}}{\sigma_{g}^{\left(c\right)}}\left(i\in S_{c}\right),$$

and a conservatively ‘consensus‐high’ score was computed as the minimum *z*‐score across patients,

$$s_{g}^{\left(c\right)}=\min_{i\in S_{c}}z_{\mathrm{ig}}^{\left(c\right)}.$$

Genes were then ranked within each cluster by $s_{g}^{\left(c\right)}$(descending). This procedure selects genes that are consistently high in every member of the cluster, guarding against spurious selection driven by a single outlying sample. The implementation loads the patient‐by‐gene table with cluster labels, computes per‐cluster *z*‐scores, ranks by the minimum‐*z* consensus and returns the Top‐K genes per cluster together with Excel exports of the unique and shared sets.

**FIGURE 7:**
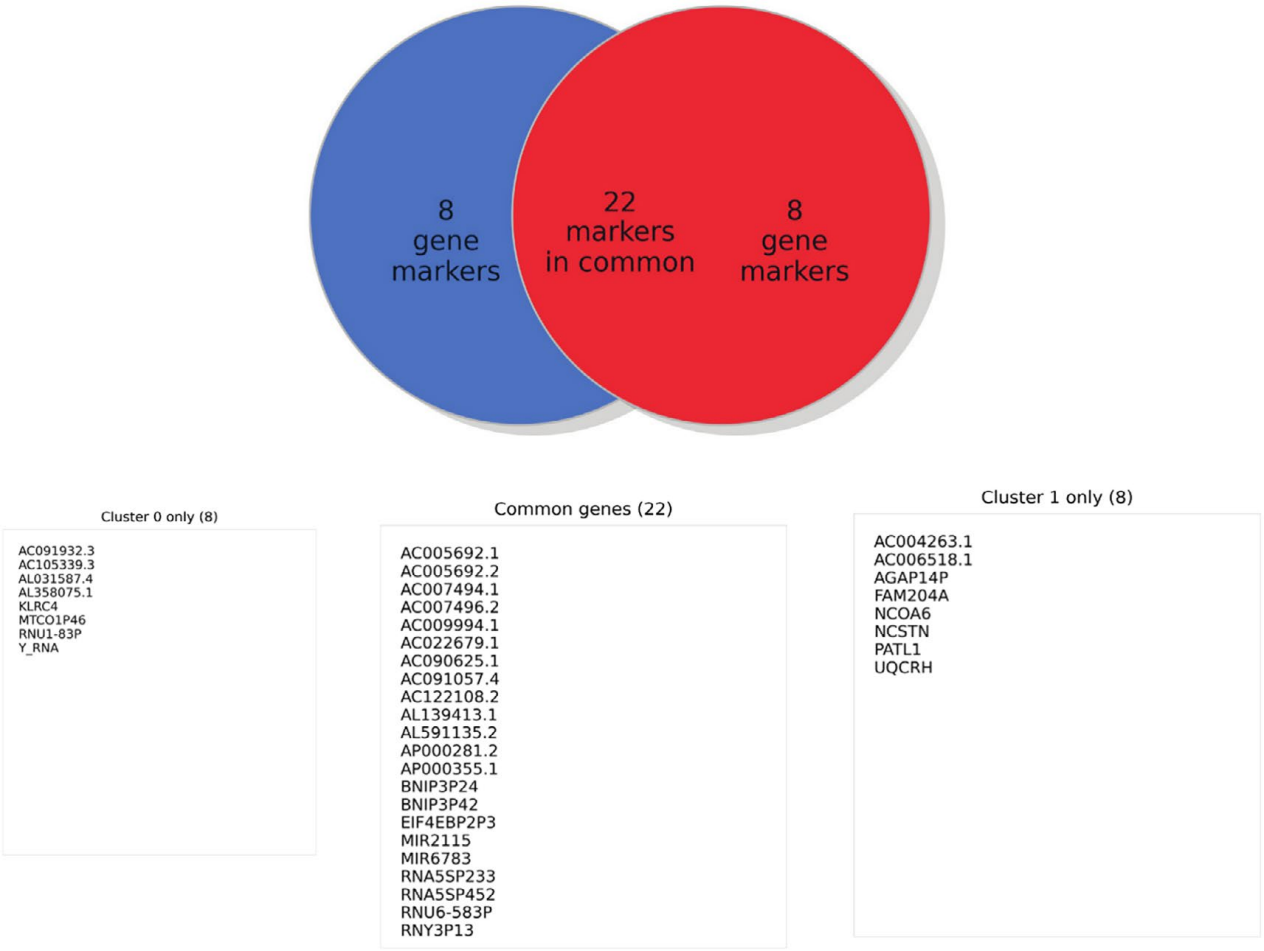
Consensus markers by cluster (Top 30 per subgroup, minimum‐*z* filter). Two‐set Venn diagram of Cluster 0 and Cluster 1 showing 22 shared genes and 8 unique genes per subgroup. Below, single‐column lists give the exact identifiers for Cluster 0‐only, Common and Cluster 1‐only gene sets. Lists and spreadsheets are generated from the consensus ranking procedure that *z*‐scores within a cluster and orders genes by the minimum patient‐wise *z*‐score.

Using *K* = 30, we obtained a compact panel comprising 22 genes common to both subgroups and 8 genes unique to each subgroup (Figure 7). The Venn diagram summarises the counts, and the three single‐column panels list the exact identifiers for Cluster 0‐only, Common and Cluster 1‐only genes. These consensus markers complement the Grad × Input–derived importance analysis by enforcing across‐patient stability inside each cluster; together, they provide a pragmatic shortlist for downstream pathway interrogation, assay design and prospective validation.

## 4 Discussion

### 4.1 Methodological Scope, External Validation and Technical Heterogeneity

A key limitation of the present study is the absence of external cohort validation. Although external validation is essential before clinical translation, it is challenging in this setting because matched glioblastoma cohorts containing whole‐slide images, RNA‐seq profiles and survival outcomes are uncommon. Moreover, both modalities are vulnerable to substantial technical heterogeneity. In digital pathology, differences in tissue processing, staining, scanner characteristics, magnification and image quality can produce domain shift across institutions. In transcriptomics, differences in library preparation, sequencing depth, gene quantification and normalisation procedures can produce protocol shift across datasets. Therefore, naïve validation using non‐harmonised cohorts may conflate biological subgroup structure with acquisition‐ or protocol‐specific batch effects. For this reason, we interpret the present framework as a hypothesis‐generating multimodal stratification approach rather than a clinically validated decision‐making tool. Prospective validation in harmonised multi‐centre cohorts with paired WSI, RNA‐seq, survival outcomes and detailed protocol metadata will be required before clinical deployment. Future validation should also include bootstrap resampling, consensus clustering and leave‐one‐out sensitivity analyses to quantify the stability of the small high‐risk subgroup.

### 4.2 Comparison With Existing Multimodal Fusion Frameworks

Compared with supervised pathomic‐fusion and attention‐based multimodal models, the present framework prioritises transparency, portability and unsupervised subtype discovery rather than direct supervised outcome prediction. Attention‐based approaches can learn complex cross‐modal interactions and may provide tile‐ or feature‐level attention maps, but they usually require larger labelled cohorts, more complex optimisation and careful control of overfitting. In contrast, our late‐fusion design compresses each modality separately, concatenates patient‐level embeddings and performs clustering without survival supervision. This simpler design is less expressive than end‐to‐end attention models, but it is easier to audit and is well aligned with discovery‐level patient stratification in a limited paired WSI/RNA‐seq cohort. Thus, the present contribution should be interpreted as a transparent unsupervised stratification framework rather than a replacement for supervised pathomic‐fusion or attention‐based prediction models.

### 4.3 Biological Characterisation of Cluster‐Specific Markers

Unbiased late‐fusion clustering yielded two prognostically distinct glioblastoma groups. To explore biological underpinnings, we examined the 8 cluster‐specific RNA markers per group.

### 4.4 Poor‐Prognosis Cluster (Cluster 1)

This set contains NCSTN (nicastrin; a γ‐secretase subunit essential for NOTCH receptor activation) and UQCRH (a Complex‐III subunit of the mitochondrial electron‐transport chain). Both point to programmes repeatedly linked with GLIOBLASTOMA aggressiveness. NOTCH signalling is upregulated in infiltrative GLIOBLASTOMA cells in vivo and is associated with invasion and treatment resistance, making the γ‐secretase complex a plausible therapeutic target [25]. Orthogonal evidence shows that GLIOBLASTOMA can rely on oxidative phosphorylation (OXPHOS); spatial proteomics revealed OXPHOS‐dominant tumour states associated with hypoxia‐adapted phenotypes, and pharmacological inhibition of respiration (e.g., Gboxin) selectively impairs GLIOBLASTOMA cells [26, 27]. Two additional protein‐coding genes, NCOA6 (a nuclear‐receptor co‐activator that amplifies oncogenic transcription) and PATL1 (a P‐body/decapping scaffold controlling mRNA stability), plausibly support stress‐tolerant, highly transcriptional states seen in aggressive gliomas [28, 29]. The remaining four markers (FAM204A, AGAP14P, AC004263.1, AC006518.1) are poorly characterised (the latter three are non‐coding/pseudogene loci); however, widespread dysregulation of long non‐coding RNAs (lncRNAs) in GLIOBLASTOMA contributes to chromatin remodelling and transcriptional re‐wiring, consistent with their appearance as cluster features [30].

### 4.5 Better‐Prognosis Cluster (Cluster 0)

This set includes KLRC4 (NKG2F; an NK‐cell receptor gene) together with several non‐coding/pseudogene loci (e.g., Y_RNA, RNU1‐83P, AC091932.3). NK‐cell‐related signatures have been associated with more favourable outcomes in glioma, consistent with a relatively ‘hotter’ immune micro‐environment in this cluster [31]. Enrichment for small non‐coding RNAs aligns with broad roles for RNA‐based regulation in tempering proliferation and shaping anti‐tumour immunity in brain tumours [32].

Overall, the poor‐prognosis cluster showed markers consistent with NOTCH/γ‐secretase and mitochondrial OXPHOS axes, whereas the better‐prognosis cluster showed immune/NK‐cell and non‐coding RNA signals. These coherent biological themes support the plausibility of the discovered subgroups. However, they should be interpreted as hypothesis‐generating signals rather than evidence of treatment sensitivity. Future experimental and clinical studies will be required to determine whether these pathways represent actionable vulnerabilities in the identified subgroups.

### 4.6 Histology‐Level Explainability

Although the present study includes RNA‐level attribution and consensus gene‐marker analyses, histology‐level explainability remains limited. WSI information was incorporated through tile‐level ResNet‐50 feature extraction followed by patient‐level aggregation and autoencoder compression. Generating reliable tile‐level heatmaps would require an additional supervised or weakly supervised explainability module, because the final subtype labels are derived from unsupervised patient‐level clustering rather than from a tile‐level classifier. In addition, biologically meaningful interpretation of highlighted regions would require pathologist review. We therefore consider tile‐level heatmaps, representative cluster‐prototype regions and pathologist‐guided assessment important future extensions rather than components of the present proof‐of‐concept study.

## 5 Conclusions and Future Work

### 5.1 Conclusions

We present a transparent workflow that fuses routine haematoxylin–eosin whole‐slide histology with bulk RNA‐seq to identify survival‐associated glioblastoma subgroups using unsupervised learning after feature extraction. The late‐fusion embedding (30‐D histology + 30‐D RNA; PCA retaining ~98% variance) produced a parsimonious two‐cluster solution with the highest internal validity among the models tested, outperforming either modality alone and remaining stable after adding simple clinical covariates. Patients in the minority cluster experienced substantially worse survival, and the cluster effect persisted after age adjustment, suggesting that the fused latent space captures prognostic signal beyond age alone. Together with RNA‐derived attributions and the consensus‐marker procedure, the analysis generated compact, interpretable gene sets aligned with plausible biological themes. However, because the poor‐prognosis cluster is small and external validation has not yet been performed, these findings should be interpreted as hypothesis‐generating rather than clinically validated. All code, configuration and figure‐generation scripts are provided to enable replication, scrutiny and adaptation to future validation cohorts.

### 5.2 Translational Outlook and Future Work

The present study should be viewed as a hypothesis‐generating framework. The following steps are required before clinical translation:

1. Prospective, harmonised multi‐centre validation. Future studies should validate the two‐subtype solution in independent cohorts with matched WSIs, RNA‐seq, survival outcomes and detailed protocol metadata. Such validation should explicitly address WSI domain shift, including scanner, staining, magnification, tissue‐processing and image‐quality differences, as well as RNA‐seq protocol shift, including library preparation, sequencing depth, quantification and normalisation differences.
2. Cluster‐stability and sensitivity analysis. Future work should quantify the stability of the minority high‐risk subgroup using bootstrap resampling, consensus clustering, leave‐one‐out or leave‐few‐out analyses and pre‐registered external validation. These analyses are particularly important because the poor‐prognosis cluster is small.
3. Assay design for minimal gene panels. The consensus and attribution analyses nominate concise gene sets. Future work should evaluate targeted assays, such as qPCR or amplicon panels, for the top‐ranked transcripts per cluster, with the goal of testing whether a low‐cost signature can recapitulate fused subtype assignment in independent cohorts.
4. Extension from prognosis to treatment response. The current framework identifies survival‐associated multimodal subgroups but does not test whether subtype assignment predicts differential response to temozolomide, radiotherapy, immunotherapy or targeted agents. This distinction is important because a prognostic biomarker is not necessarily a predictive treatment‐response biomarker. Future studies should therefore evaluate subtype‐by‐treatment interactions in cohorts with harmonised treatment exposure, progression, recurrence, radiological response and survival annotations. Similar strategies have been used in other cancers, where molecular or tumour‐microenvironment signatures were extended from prognostic stratification towards immunotherapy or personalised treatment‐response assessment [33, 34]. For glioblastoma, this would require testing whether the identified high‐risk cluster is enriched for resistance‐associated pathways and whether NOTCH/γ‐secretase or OXPHOS‐associated features correlate with experimentally measured drug sensitivity in patient‐derived models.
5. Research‐grade pipeline and future decision support. The current pipeline should be considered a research‐grade framework. Future versions may be developed into clinical decision‐support tools only after external validation, calibration, treatment‐response assessment, pathologist‐guided histology explainability and prospective testing.
6. Biological validation. Orthogonal assays, including IHC for NOTCH/OXPHOS markers, multiplex imaging for immune/NK‐cell features and perturbation studies in patient‐derived models, will be required to confirm pathway activity in each subgroup and test whether the observed signatures correspond to functional vulnerabilities.

PMID: 36899891 is titled ‘The Characteristics of Tumor Microenvironment Predict Survival and Response to Immunotherapy in Adrenocortical Carcinomas,’ and PMID: 36358764 is titled ‘A Novel m7G‐Related Genes‐Based Signature with Prognostic Value and Predictive Ability to Select Patients Responsive to Personalized Treatment Strategies in Bladder Cancer.’

## 6 Limitations

The principal limitation of this study is the small size of the poor‐prognosis cluster (*n* = 8 in survival analyses). This subgroup may represent a rare, clinically important high‐risk phenotype, but the small sample size limits generalisability, reduces precision of survival estimates and increases the possibility that the cluster reflects cohort imbalance or outlier structure. Therefore, the minority cluster should be interpreted as a candidate subgroup requiring validation rather than a clinically established glioblastoma subtype.

A second major limitation is the absence of external validation. Although external validation is essential, matched glioblastoma cohorts containing whole‐slide images, RNA‐seq profiles, survival outcomes and adequate protocol metadata are uncommon. In addition, external validation across non‐harmonised datasets can be confounded by WSI domain shift and RNA‐seq protocol shift. Differences in tissue processing, staining, scanning, magnification and slide quality may alter histology embeddings, while differences in library preparation, sequencing depth, gene quantification and normalisation may alter transcriptomic embeddings. These factors could cause a model to learn dataset‐specific technical variation rather than reproducible tumour biology.

Third, survival modelling was adjusted for age, but additional clinical and molecular variables such as MGMT promoter methylation, IDH status, treatment details, extent of resection and performance status were not uniformly available in the paired cohort. Given the very small minority cluster, heavily adjusted Cox models would also be statistically unstable and prone to overfitting. Finally, the present study does not test treatment‐response prediction and does not include tile‐level histology explainability. These limitations motivate future work involving harmonised multi‐centre validation, bootstrap and consensus‐clustering stability analysis, richer clinical covariates, subtype‐by‐treatment interaction testing, representative WSI‐region analysis and pathologist‐guided validation.

In summary, auditable late fusion of WSIs and RNA‐seq identified two biologically plausible and survival‐associated glioblastoma subgroups from retrospective routine data. The framework provides a transparent basis for hypothesis generation, pathway prioritisation and future validation studies. However, because the poor‐prognosis cluster is small and external validation has not yet been performed, the proposed subgroups should not be interpreted as clinically validated decision‐making categories. Harmonised multi‐centre validation, treatment‐response analysis, cluster‐stability assessment and histology‐level explainability will be required before this approach can be translated into clinical decision support.

## Author Contributions

**Amin Zadeh Shirazi:** conceptualisation, investigation, methodology, data curation, visualisation, writing – original draft, formal analysis. **Guillermo A. Gomez:** conceptualisation, investigation, funding acquisition, writing – review and editing, validation, methodology, formal analysis, project administration, supervision, data curation.

## Funding

This work was supported by a McCleary Murchland Fellowship [to G.A.G.]; grants from the NHMRC (Ideas grant 2021/GNT2013180 and 2023/GNT2030541 to G.A.G.); the Neurosurgical Research Foundation [to G.A.G. and A.Z.S]; the Charlie Teo Foundation [Rebel Grant to G.A.G.]; Tour de Cure Grants to [G.A.G].

## Ethics Statement

This study used de‐identified, publicly available data from The Cancer Genome Atlas (TCGA) and did not involve prospective recruitment of human participants, new collection of identifiable patient data or animal experimentation.

## Consent

The authors have nothing to report.

## Conflicts of Interest

The authors declare no conflicts of interest.

## Data Availability

Publicly available datasets were analysed in this study. Whole‐slide images and RNA sequencing data were obtained from The Cancer Genome Atlas (TCGA) via the NCI Genomic Data Commons (GDC) portal. The code used for preprocessing, feature extraction, multimodal integration, clustering and survival analysis is available from the authors upon request.
